# Clinical Management of Children and Adolescents with Neurofibromatosis Type 1 Like Phenotypes and Complex Behavioural Manifestations: A Multidisciplinary and Dimensional Approach

**DOI:** 10.1155/2019/4764031

**Published:** 2019-12-31

**Authors:** Ana Moscoso, Aurélie Julien, Antoine Tanet, Angèle Consoli, Martine Pagnard, France Trevisan, Isabelle Kemlin, Diana Rodriguez, David Cohen

**Affiliations:** ^1^Department of Child and Adolescent Psychiatry, Robert Debré Hospital, Paris, France; ^2^Department of Child and Adolescent Psychiatry, Reference Center for Rare Psychiatric Diseases, APHP, Groupe Hospitalier Pitié-Salpêtrière, Université Sorbonne, Paris, France; ^3^GRC-15, Approche Dimensionnelle des Épisodes Psychotiques de L'enfant et de L'adolescent, Faculté de Médecine, UPMC, Sorbonne Universités, Paris, France; ^4^Centre de Référence des Neurofibromatoses et Service de Neurologie Pédiatrique, AP-HP, Hôpital Armand Trousseau, GHUEP, Paris, France; ^5^Sorbonne Université, Hôpital Armand Trousseau, F-75012, Paris, France; ^6^Institut des Systèmes Intelligents et Robotiques, CNRS, UMR 7222, Université Sorbonne, Paris, France

## Abstract

*Introduction.* Cognitive and behavioural problems associated with Neurofibromatosis type 1 (NF1) are common sources of distress and the reasons behind seeking help. Here we describe patients with NF1 or NF1-like phenotypes referred to a Tier 3 Child and Adolescent Psychiatry Department and highlight the benefits of a multidisciplinary assessment.

**Methods:**

Prospective data were gathered from NF1 patients aged 7–15 years, referred by the NF1 Referral Centre due to additional difficulties either in management or diagnosis. For the selected cases, we performed a psychiatric assessment, a tailored neuropsychological evaluation based on clinical demands and history, broad speech and motor skills evaluations if there were concerns regarding language, motor abilities and/or learning difficulties and autism specific evaluations, if clinically relevant. No exclusion criteria were applied.

**Results:**

Complex NF1 cases represented only 5% of the patients (11/224). Assessments revealed the complexity of NF1 phenotype and a variety of problems including learning difficulties, emotional problems and autism spectrum disorders. Specific evaluations of language, motor, attentional and neurovisual domains were essential to guide tailored intervention strategies.

**Conclusions:**

In terms of clinical implications, the heterogeneity of NF1 phenotypical manifestations needs to be considered when developing assessment and remediation approaches for children with complex NF1.

## 1. Background

Neurofibromatosis type 1 (NF1) affects approximately 1/3,000 individuals worldwide [1]. Diagnostic criteria are varied and include *café-au-lait* spots, neurofibromas, freckling of the axillary or inguinal regions, optic glioma, Lisch nodules, distinctive osseous lesions (such as sphenoid dysplasia or thinning of long bone cortex), as well as having a first-degree relative evidencing NF1 symptoms as detailed above [2].

Although NF1 is an autosomal dominant condition (familiar type), de novo mutations account for up to 50% of the cases (sporadic type). *NF1* is completely penetrant, nonetheless it exhibits variable clinical expressivity, even between family members with the same *NF1* mutation [3]. Most *NF1* mutations result in reduced intracellular levels of the protein neurofibromin, leading to excessive cell proliferation, including development of neurofibromas and tumours and diminished cognitive capacity [4].

In clinical practice, cognitive and behavioural problems associated with NF1 and their impact on academic performance is a common source of distress and the reason behind seeking help in child psychiatry. Indeed, between 30% and 70% of individuals with NF1 have learning disabilities concerning speech, reading, writing, spelling and mathematics. These problems represent the most significant cause of lifetime morbidity associated with the disease [5, 6]. Even though some phenotypic patterns have been suggested in the past, a more in-depth analysis reinforces the need to tailor the diagnosis and the treatment of these patients.

The global cognitive functioning of NF1 patients is usually preserved, although somewhat lower when compared to unaffected siblings or peer groups [7]. Intellectual disability, defined clinically as an intelligence quotient (IQ) lower than 70 in cognitive evaluations, is estimated to be around 4–8% in NF1 patients vs. 2-3% in the general population [8, 9]. However, the exclusion of patients with central nervous system (CNS) pathology from cohort studies of NF1 prevalence reduces the number of patients with clinically moderate to severe NF1 [8] and may underestimate the prevalence of intellectual disability amongst this population.

For many years the Child and Adolescent Psychiatry Department of Pitié-Salpêtrière has been treating paediatric patients who present neuropsychiatric symptoms of rare diseases [10–12] as examples. As a specialised team, the department receives complex patients which present significant management issues. NF1 patients are referred to this department. Here we describe patients with NF1 received in our Departement and highlight the benefits of a multidisciplinary assessment.

## 2. Methods

### 2.1. Patients

Prospective data were gathered from NF1 patients (NIH, 1988) aged 7–15 years, referred by the Neurofibromatosis Referral Centre at Trousseau Paediatric Hospital for evaluation at the Department of Child and Adolescent Psychiatry at University Hospital La Pitié-Salpêtrière. The data spans from 2009 until 2016. The procedures regarding the assessments and follow-ups at the Referral Centre are described elsewhere [13]. Referrals were made due to additional difficulties either in management or diagnosis of patients and represented only a small part of the cohort (11/224—approximately 5%). No exclusion criteria were applied as we wanted to describe all complex aspects of this clinical sub sample.

### 2.2. Procedures and Instruments

Patients were assessed in medical consultations and further comprehensive evaluations. For the selected cases, we extracted from the prospective database: sociodemographic data (age, sex, academic level); all relevant information from the semi-structured medical interview to evaluate the patient's personality and family history of psychiatric and medical disorders, including NF1 clinical features, complications and follow-ups; and all relevant biological (e.g. genetic testing), physiological (e.g. electro-encephalography) and imaging (e.g. magnetic resonance imaging) information. In addition, we performed (1) a psychiatric assessment; (2) a tailored neuropsychological evaluation (e.g. executive function, attention, memory, neurovisual) based on clinical demands and history; (3) a broad speech evaluation, if there were language (oral and/or written) concerns or learning difficulties; (4) a global and fine motor skills evaluation, if there were concerns regarding motor abilities and/or learning difficulties (such as difficulties to write), and (5) autism specific evaluations, if clinically relevant. The list of testing is given in Tables 1 and 2.

Evaluations were performed because they had clinical relevance. They were adapted to the needs and characteristics of each patient. Medical files containing clinical data were thoroughly explored (AM, AJ, MP) and relevant data were included (AM, AJ, AT, MP). Psychiatric diagnoses were made according to DSM-5 criteria. Finally, the scores obtained in neuropsychological, speech and fine motor skills assessments were converted into standard deviations in order to have unique common statistics and to ease presentation.

## 3. Results

Eleven patients were included. All, except one, were boys with a mean age of 10.6 (range 7–15) years. Five patients had a first degree relative with NF1 (father, mother or sibling). Two patients lived in foster care and five with one parent (due to divorce). One had Legius syndrome, a genetic condition associated with *sprouty related EVH1 domain containing 1 (SPREAD1) *gene mutation, which phenotype overlaps that of NF1 [14]. For clarity, we present the patients in two separate tables.  Table 1 summarises the clinical profile of four NF1 patients with an intellectual deficiency and Table 2 presents the profile of patients with subnormal IQ.

### 3.1. Reasons for Referral

Patients came with multiple and diverse complaints. Reasons for referral included learning difficulties (due to the consequences of instrumental difficulties, suspicion of attention deficit hyperactivity disorder (ADHD) or intellectual disability; *N* = 7), or emotional problems or suspicion of emergent psychopathology (*N* = 6). A few (*N* = 4) were referred because of autism spectrum disorder (ASD) (doubts regarding diagnosis or difficulties in clinical management).

### 3.2. NF1 Clinical Characteristics and Intellectual Impairment

Clinical complications of NF1 (skeletal, vascular, malignancy, epilepsy, precocious puberty, the presence of unidentified bright objects (UBOs) in CNS) were present in patients who also had a certain degree of intellectual disability (Table 1), sometimes accompanied by autistic features (patients 1, 2, 4; Table 1). Only, one patient with NF1 complications did not present with an intellectual disability but had a borderline IQ (patient 8; Table 2). In four patients, UBOs were present mainly in the cerebellum and the basal ganglia (patients 1, 2, 4 and 8). NF1 complications motivated frequent medical appointments and hospitalisations, an additional burden for patients and their families. For all the other patients (Table 2) only mild NF1 features, such as café-au-laits spots, intertriginous freckling, few neurofibromas and/or Lisch nodules, were present. These patients all had subnormal IQ.

### 3.3. NF1 and Learning Disabilities

All patients had learning disabilities that appeared as a primary or secondary cause of referral. For the patients who had subnormal IQ scores (Table 2), it was common to find discrepancies in IQ sub-scales. Within each subscale, scores could vary widely (data not shown). Even if the patients received an* average/mean* IQ, their global intellectual efficiency was weaker when compared to the general population.

Specific learning disabilities were also common. They included specific language developmental delays (patients 5, 6, 9, 11; Table 2), graphomotor delays (patients 5, 6, 7, 8; Table 2) and developmental coordination disorders (dyspraxia) (patients 8, 9; Table 2). Some patients also had comorbid ADHD (patients 5, 6, 8; Table 2). The extensive developmental evaluation was crucial to disentangle diagnosis and also to guide further therapeutic propositions. Such specific propositions included school adaptations (such as having more time to finish evaluations, the use of computer to write), speech and reading therapies once or twice per week, and motor skills remediation once per week, that lead to improvement (nonpharmacological treatment; Table 2).

Patients with intellectual disabilities (Table 1) arguably presented learning difficulties. Standard tests for cognitive evaluation (such as the Wechsler intelligence scale for children, WISC [29]) were difficult to perform in such patients. In these cases, clinical evaluation and developmental scales were helpful (Brunet Lezine [18]), Terman Merrill [25]), as well as some psychosocial scales (Psychoeducational Profile (PEP) [23], Vineland [28]) that estimate performance in more ecologic day to day activities. In this context, specific evaluations of language, motor, and attentional domains are equally important to guide tailored intervention strategies. Intervention strategies were generally more global in the context of schooling for disabled children.

### 3.4. NF1 and Attentional Issues

Four of our patients had an ADHD diagnosis, obtained after clinical assessment and a specific neuropsychological evaluation (Test of Everyday Attention for Children, TEA-Ch [24]). A fifth patient (patient 4; Table 1) diagnosed with ADHD was evaluated through clinical observation and functional scales (Conners [20]), because the neuropsychological evaluation was difficult to perform given the intellectual deficit. In all cases, an ADHD diagnosis was made along with other comorbidities and was never isolated. Four patients were treated with methylphenidate and one was given specific cognitive training addressing attention. Three patients receiving methylphenidate improved and one stopped the treatment due to side effects.

### 3.5. NF1, Emotional Problems and Emergent Psychopathology

The most frequent diagnosis was anxiety disorder and/or NF1 related stress (patients 1, 4, 7, 10, 11). Two visited for the evaluation and treatment of mood disturbances (patients 8, 11; Table 2). Finally, another patient visited for psychiatric evaluation in the context of sexual assault (patient 3; Table 1). Medication along with nonpharmacological treatments such as different types of psychotherapy were proposed and appeared to be helpful.

### 3.6. NF1 and Autistic Spectrum Symptomatology

Four patients presented clear autistic features after clinically comprehensive/developmental assessment and additional assessment tools (patients 1, 2, 4; Table 1 and patient 9, Table 2). Three patients with autistic features had concurrent intellectual disability (Table 1). A fifth patient with an ongoing history of social difficulties had a pragmatic communication disorder (patient 5; Table 2). Complementary psychological tests were helpful for the diagnosis (CARS [19], ADI [15], ADOS [16]). Treatment strategies addressed co-morbidities as well as core features of autism (e.g., social strategies in group) in outpatient settings or in the context of specific schooling models.

## 4. Discussion

This case series reflects the variety of problems and the complexity of severe NF1 paediatric patients. The gender bias reflected in our sample may result from the overrepresentation of boys in child and adolescent psychiatry consultations and therefore will not necessarily reflect the epidemiology of NF1 [30]. Also, the morbimortality of the disease, is mild to moderate in most cases. Nonetheless, clinical complications of NF1 can present a serious burden. This was the case for most of the patients reported here, who were referred to a special psychiatric clinic for rare diseases. The large heterogeneous phenotypic expression of NF1 is likely to be a consequence of the stochastics chain of events associated with NF1. At a molecular level, the reduced intracellular levels of neurofibromin found in NF1 patients induce impairments in learning and memory through imprecise, i.e. abnormally high or low RAS modulation and consequential gamma-aminobutyric acid (GABA)-mediated excessive inhibition in the hippocampus [4, 31]. Also, patients with NF1 may show subcortical unidentified bright objects (UBOs) as we found in four of our patients. In all but one, UBOs were present in patients with intellectual disability and a multitude of somatic complications of the disease. UBOs are present in approximately 60–70% [32, 33] of the children with NF1 but tend to disappear with age. Histopathologic studies have shown that UBOs correspond to areas of myelin vacuolization with increased water content [34] and therefore, could reflect disordered myelination [35]. The presence of UBOs has been associated with lower intellectual ability [36, 37] and also visuospatial impairments when UBOs were located in the cerebellum [38]. The latter seems to be the case for patients 1 and 2 (Table 1) and patient 8 (Table 2) in our case series.

### 4.1. NF1 Cognitive Impairment and Learning Disabilities

In this case series, the patients presented either a degree of intellectual disability (Table 1) or an average but heterogeneous cognitive profile with significant functional impairments (Table 2). Many studies have now made clear how intelligence is only mildly affected in the vast majority of NF1 patients. However, specific impairments in cognition are very common (up to 80% of children in NF1 clinics [8]) and have a negative impact in the quality of life. Some specific deficits have been reported to NF1: visual-spatial deficits [39], speech and language deficits, motor skill deficits [40], social skill deficits [5, 41] and attentional deficits [8, 42]. All may lead to learning disabilities and further emotional suffering. The presence of visual-spatial and attentional deficits has been robustly replicated [7, 43]. However, research has revealed contradictory results regarding motor skills and language performance in patients with NF1 [7]. Moderate to severe intellectual disability and severe clinical cases seemed to be the focus of interest of early works in neurofibromatosis [44] but severe cases of NF1 have received little attention recently.

### 4.2. NF1 and ADHD

In all our NF1-ADHD patients, the ADHD diagnosis was made along with other comorbidities and was never isolated. Patients with NF1 present more symptoms and are more often diagnosed with ADHD than the general population. Prevalence estimates range from 30% to 50% [45, 46], which are higher than those expected in the general population, i.e. about 5% in children and 2.5% in adults [47, 48].

Clinical and cognitive profiles of both ADHD patients with and without NF1 are heterogeneous. Individuals are affected in different domains of attention, impulsivity, hyperactivity and executive functioning, and to different degrees [46, 49]. Visual attention seems consistently impaired in NF1 patients [50, 51] and would lead to instability in focusing attention and lower resistance to interference in controlled tasks but also to inattentive and impulsive behaviour in natural environments.

Importantly, ADHD-NF1 patients seem responsive to methylphenidate [42, 46]. This improvement has been also reported for children with IQs lower than 80 [46]. In our case series, four of the patients diagnosed with ADHD were treated with methylphenidate, three showing a great improvement and one dropping out of treatment due to side effects.

The high frequency of ADHD in children with NF1, as well as the demonstrated significant comorbidity of ADHD with literacy learning disabilities [8] and social skills problems [52] indicates the need for thorough screening of ADHD symptomatology in all children with NF1 [45]. Besides, the impact of attention deficits and behaviour problems in children with NF1 often leads to lower social acceptance and lack of self-confidence/esteem. When emotional problems are secondary to ADHD, effective treatment of ADHD can improve them both [46].

### 4.3. NF1 and Autistic Spectrum Symptomatology

After some early work evoking associations between autism and NF1 [53] there is a re-enacted concern regarding ASD and NF1. Since Huijbregts and de Sonneville [54] showed that NF1 impairments in cognitive control (i.e. a combination of processing speed, working memory, inhibitory control, and emotional processing functions) is associated with the presence of autistic traits, several studies have explored the prevalence of ASD symptoms in NF1 populations.

**Figure 1:**
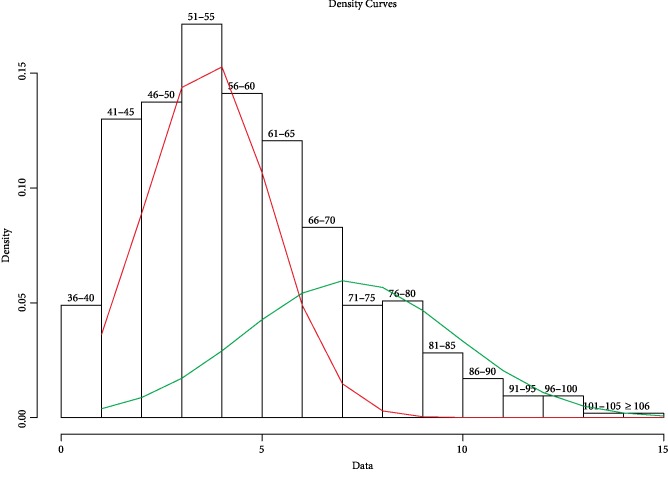
Theoretical distribution of social responsiveness scale (SRS) scores in individuals with NF1 (*n* = 412).The admixture analysis shows that the best fitting model for SRS scores in NF1 founds 2 subgroups with low and high scores (mean SRS = 55 (red curve) and 70 (green curve), respectively). Data from Morris et al. [59].

Studies focusing on social impairments as a continuous variable within the social responsiveness scale (SRS) [55–58] showed that NF1 patients present more social impairments than the general population (up to 40% in some samples [56]). Whether or not this is enough to state that ASD is increased in NF1 patients is another question. First, ASD screening tools are not meant to provide a diagnosis of ASD, but rather identify children who should be further evaluated. Second, NF1 patients with high SRS scores show mild to moderate ASD symptomatology (e.g. latter diagnosis during school years; no stereotyped behaviours) [57]. We believe that research should explore further the meaning of ASD symptomatology in NF1. Given the rarity of stereotyped behaviours in NF1, perhaps the DSM-5 diagnosis of social communication disorder will be more appropriate in some cases? Also, is it legitimate to use SRS score as a continuous variable? Indeed, when re-analysing the largest cohort of NF1 so far that reported SRS scores [59] we found that the best model to predict data distribution using admixture analysis was a bimodal distribution (Figure 1), with a large group of patients with a mean SRS score equal to 55 (red curve), and a smaller group of patients with a higher mean SRS score equal to 70 (green curve). This means that considering SRS as a continuous variable showing a normal distribution may be inappropriate. This also means that NF1 phenotype heterogeneity might be better understood separating subgroups of patients as it has been shown in other genetically complex neurodevelopmental disorders.

However, we do believe that rare typical cases of ASD can also be encountered in NF1. Three patients in this case series presented an unequivocal profile of ASD comorbid with intellectual disability. Several authors postulated that autism associated with neurogenetic syndromes can be classified as “complex autism” or “syndromic autism” [60–62]. Complex autism is characterised by lower IQ, higher rates of comorbidities both psychiatric and somatic, and higher rates of epilepsy [63, 64]. For these severe cases, this classification may be useful to better explore their clinical profile and tailor treatment to the child's needs [65].

### 4.4. NF1 and Emotional Problems

NF1 has also significant social and psychological consequences on individuals and their families [66, 67]. Most patients of this case series were referred with emotional problems. While some (*n* = 7) had behavioural difficulties (sometimes secondary to ADHD or to learning disabilities), others expressed internalising symptoms such as anxiety or depression. These conditions can often be found in NF1 [67]. The emotional impact of cosmetic deformities, the fear of malignancy, and the medical complications, such as the management of hypertension or the need for surgical interventions should be carefully evaluated and not be neglected. Learning difficulties can also generate feelings of low self-esteem and inefficiency that can last in time. In addition, the impact of attention deficits and behaviour problems in children with NF1 often leads to lower social acceptance and lack of self-confidence. As mentioned previously, when emotional problems are secondary to ADHD, effective treatment of ADHD can improve them both [46]. Besides, parents of NF1 children experience greater stress than other parents [68], sometimes associated with feelings of guilt regarding the genetic transmission of NF1. The parents should also receive proper care.

### 4.5. Treatment Modalities and Outcome

Strategies that decrease either RAS activity or GABA-mediated inhibition have been suggested to specifically treat learning and emotional deficits associated with NF1. Pharmacologically targeting this pathway with an HMG-CoA reductase inhibitor (an anti-RAS agent), such as lovastatin [69] evidenced some encouraging results in animal models. However, to date, clinical trials with NF1 patients have shown modest results [70–72]. Therefore, current treatment options remain symptomatic. The heterogeneity of NF1 phenotypical manifestations needs to be considered when developing assessment and remediation approaches for children with NF1. The outcome is therefore variable, different for each patient.

## 5. Conclusions

In severe cases of NF1 with important psychiatric morbidity, it can sometimes be difficult to disentangle specific domains of impairment, as cognition as a whole is complex. In day-to-day activities, more than one cognitive domain is needed to perform adequately, making the task of disentanglement even more challenging. This is why a multidimensional and longitudinal clinical evaluation is warranted when assessing patients.

Phenotype-genotype correlations tend to subscribe the idea that behavioural manifestations present themselves with continuous variation. This type of pattern was proposed for NF1 autistic symptoms [59]. However, we believe that more complex phenotype/genotype association occurs in NF1 for psychiatric comorbidity including ASD symptomatology. Similarly to other genetic conditions such as juvenile myotonic dystrophy [73], or tuberous sclerosis [74], bimodal phenotypic patterns of intelligence/cognitive performance seem to adapt better to the distribution of IQ scores in these populations. In terms of clinical implications, the heterogeneity of NF1 phenotypical manifestations needs to be considered when developing assessment and remediation approaches for children with NF1.

## Figures and Tables

**Table 1:** Clinical profile of NF1 patients with an intellectual deficiency.

	Patient 1	Patient 2	Patient 3	Patient 4
Sex, age	Male, 12 years	Male, 13 years	Male, 15 years	Female, 8 years

Main reasons for referral	Increasing puberty-onset aggressiveness	ASD assessment	Psychiatric assessment in the context of sexual assault	Suspicion of ADHD
	Suspicion of ADHD	Suspicion of ADHD	Outpatient unit	Social problems (foster care)
	Inpatient unit	Outpatient unit		Outpatient unit

NF1 diagnosis	Familial NF1 (including intellectual disability)	Sporadic NF1	Familial NF1	Familial NF1
		Additional genetic research was negative: karyotype, x fragile		

NF1 complications	Dystrophic thoracic scoliosis	Sphenoid bone dysplasia	Bilateral optic pathway tumor, remission	Optic pathway tumor, remission
	Sphenoid bone dysplasia	Orbicular-facial plexiform neurofibroma (surgically removed)		Precocious puberty
	Labile renovascular hypertension	UBOs**:** basal ganglia, cerebellum		Epilepsy
	Intermittent claudication			UBOs: left pallidum, white matter
	Several complicated surgical interventions/hospitalisations			
	Absence of pheocromocytoma or precocious puberty			
	UBOs: bilateral temporal; right lentiform nucleus; cerebellum/protuberance			
			Blindness	

Developmental delay (early history)	Vineland (age 4): developmental delays ranging from 7–39 months	Hypoxia at birth	?? (family poorly informative)	Prematurity
	(Repeated gastroenteritis during childhood)	(Repeated gastroenteritis during childhood)		PEP-R (age 3): Average developmental delay 11–13 months

Cognitive assessment	TERMAN–MERRILL	PEP-3: mild intellectual disability	Assessment not available	BRUNET-LEZINE (age 3): Developmental delays ranging from 8–14 months
	Moderate intellectual disability	Divided attention TEA-Ch: −2.6SD		
		Unimodal attention TEA-Ch −2.6SD		
		Language EXALANG: delays in all domains (oral/written) (average: −1.7SD)		
		Flexibility NEPSY-II: −1.4SD		
		Working memory NEPSY-II: −1.4SD		

ASD assessment	CARS (age 4) = 30.5 (mild autism)	Clinical evaluation: repetitive behaviors and perseverant thoughts	No clinical suspicion	ADOS
	ADI-R: stereotypies = 9 (threshold 3)	Deficits in social cognition/pragmatics: −1.3SD ^EMOTION COMPREHENSION TEST^		Communication domain = 6 (threshold 4)
	Communication domain = 14 (threshold 8)			Social interaction = 14 (threshold 7)
	Other: anxiety			

Psychiatric diagnosis	Moderate intellectual disability	Mild intellectual disability	Mild intellectual disability	Moderate intellectual disability
	Autism	Autism		Autism
	Dyspraxia	Dyspraxia		Dyspraxia
	NF1 related stress (comorbidities)	ADHD-hyperkinetic type		NF1 related stress (comorbidities)
	No criteria for ADHD			ADHD-hyperkinetic type ^CONNERS; clinical assessment^

Medication	Aripiprazole	Methylphenidate	None	Levetiracetam
	Melatonin	Melatonin		Enantone
	Labetalol			Methylphenidate
	Clonidine			Risperidone
				Melatonin

Nonpharmacological treatment	Full time school for disabled adolescents, including remediation to improve attention, speech, motricity	Full time school for autistic adolescents	Full time school for disabled adolescents	Full time school for disabled children, including remediation to improve attention, speech, motricity.
	Regular follow-up	Adapted school activities, including remediation to improve attention	Regular psychiatric follow up	Foster family
	Regular multidisciplinary discussion	Speech remediation in the past		

Outcome	Clinical improvement	Stable	Improvement	Improvement (less than expected due to social difficulties)
	Balancing day-to-day life with the ongoing co-morbidities of NF1 is a challenge to him and his family			Balancing day-to-day life with the ongoing co-morbidities of NF1 is a challenge to her

ADHD, attention deficit hyperactivity disorder; ADI-R, autism diagnostic interview-Revised [15]; ADOS, autism diagnostic observation schedule [16]; ASD, autism spectrum disorder; BRUNET-LEZINE, First childhood psychomotor developmental schedule [18]; CARS, The childhood autistic rating scale [19]; CONNERS, Conner's continuous performance test [20]; EMOTION COMPREHENSION TEST, Test of emotion comprehension [27]; EXALANG, language evaluation for children 8–11 years [21]; NEPSY-II, Neuropsychological test for children [22]; NF1, Neurofibromatosis type 1; PEP, Psychoeducational profile: Revised (PEP-R) and third edition (PEP-3) [23]; TEA-Ch, test of everyday attention for children [24]; TERMAN-MERRILL, Stanford-Binet intelligence scale [25]; UBOs, unidentified bright objects; VINELAND, adaptive behaviour scales [28].

**Table 2:** Clinical profile of NF1 patients with subnormal IQ.

	Patient 5	Patient 6	Patient 7	Patient 8	Patient 9	Patient 10	Patient 11
Sex, age	Male, 10 years	Male, 8 years	Male, 8 years	Male, 9 years	Male, 15 years	Male, 7 years	Male, 12 years

Main reason for referral	Learning difficulties	Learning difficulties	Learning difficulties	Intermittent explosive behaviour; ADHD	Confirmation of ASD (ADOS-ADI-R)	Suspicion of ADHD	Mood disorder
	Anxiety symptoms	Behavioural difficulties	Hyperactivity sleep; family related issues	NF1 complications: Precocious puberty; nondysplastic scoliosis; UBOs: cerebellum, *globus pallidus*	Emotional lability	NF1 related stress	Learning difficulties
	Social difficulties						
	Outpatient unit	Outpatient unit	Outpatient unit	Outpatient unit	Outpatient unit	Outpatient unit	Outpatient unit

NF1 diagnosis	Familial NF1	Familial NF1	Sporadic NF1	Sporadic NF1	Sporadic NF1	Sporadic NF1	Legius SD

Developmental delay	Minor (graphic abilities)	No	Minor (global motor)	Walking unstable at 21 months	???	No	Not reported
	Repeated otitis		Prematurity	Speech delay (Repeated otitis)			

Cognitive assessment WISC-IV	Verbal: 92; performance: 121; working memory: 97; speed: 88	Verbal: 86; performance: 77; working memory: 73; speed: 71	Verbal: 92; performance: 94; working memory: 76; speed: 78	Verbal: 98; performance: 65; working memory: 82; speed: 83	Verbal: 86; performance: 73; working memory: 109; speed: 100	Verbal: 92; performance: 109; working memory: 91; speed: 103	Verbal: 94; performance: 90; working memory: 73; speed: 66
	Heterogeneous	Heterogeneous	Heterogeneous	Heterogeneous	Heterogeneous	Heterogeneous	Heterogeneous

Attention and executive functions	Divided attention ^TEA-Ch^: −2SD	Divided attention ^TEA-Ch^: −1.4SD	Divided attention ^TEA-Ch^: Normal	Divided attention ^TEA-Ch^: −0.5SD		Divided attention ^TEA-Ch^: +0.7SD	
	Unimodal attention ^TEA-Ch^: Normal	Unimodal attention ^TEA-Ch^: −1.4SD	Unimodal attention ^TEA-Ch^: Normal		Unimodal attention ^TEA-Ch^: −0.7SD	Unimodal attention: ^TEA-Ch^ +0.3SD	Unimodal attention ^TEA-Ch^: −0.7SD
		Sustained attention ^TEA-Ch^: −1.7SD					
		Attention control ^TEA-Ch^: -2.2SD	Attention control ^TEA-Ch^: +0.7SD				
		Inhibition ^TEA-Ch^: −2SD				Inhibition ^TEA-Ch^: +1SD	
	Flexibility ^NEPSY-II^: −0.1SD	Flexibility ^NEPSY-II^: −1.5SD	Flexibility ^NEPSY-II^: −0.5SD	Flexibility ^NEPSY-II^: −1.5SD	Flexibility ^NEPSY-II^: −1.4SD	Flexibility ^NEPSY-II^: −0.1SD	Flexibility ^NEPSY-II^: −0.2SD

Memory	Short-term ^NEPSY-II^: −0.1SD	Short-term ^NEPSY-II^: −1.2SD	Short-term: −1.6SD		Short-term ^NEPSY-II^: −0.3SD	Short-term ^NEPSY-II^: −0.7SD	Short-term ^NEPSY-II^: −0.3SD
	Episodic^ REY^: −0.3SD	Episodic^ REY^: +0.5SD				Episodic^ REY^: +0.4SD	Episodic^ REY^: −3SD

Language assessment EXALANG	Oral receptive: -0.7SD	Oral receptive: −1.5SD	Oral expressive: +1.4SD	Normal	Normal	Normal	Oral lexicon: −1.3SD
	Oral expressive: −0.5SD	Oral expressive: −2SD	Oral lexicon: −0.5SD				
	Oral lexicon: +0.7SD	Oral lexicon: −1.2SD					
	Written receptive: −2.6SD	Written expressive: −1.7SD	Written receptive: +1.4S				Written expressive: −2.6
	Written expressive: −2.9SD		Written expressive: −0.5SD				
	Spelling: −2.6SD						

Motricity	Visuo-construction^ REY^: +0.5SD	Visuo-construction^ REY^: Normal	Visuo-construction^ REY^: −0.7SD	Visuo-construction^ REY^: −3SD	Visuo-construction^ REY^: −0.3SD	Visuo-construction^ REY^: +0.2SD	Visuo-construction^ REY^: −0.7SD
	Visuo-spatial ^NEPSY-II^: +0.5SD	Visuo-spatial ^NEPSY-II^: −0.6SD	Visuo-spatial ^NEPSY-II^: −0.3SD	Visuo-spatial ^NEPSY-II^: −0.1SD	Visuo-spatial ^NEPSY-II^: −0.8SD	Visuo-spatial ^NEPSY-II^: +0.3SD	Visuo-spatial ^NEPSY-II^: −0.8SD
	Graphomotor skill^ BHK^: −2.3SD	Graphomotor skill^ BHK^: −2SD	Graphomotor skill^ BHK^: −2SD	Graphomotor skill^ BHK^: −2SD	Graphomotor skill^ BHK^: +1.4SD	Graphomotor skill^ BHK^: +0.4SD	Graphomotor skill ^BHK^: −0.3SD

Socio-cognition/pragmatic	−0.7SD	−0.3SD			−2.2SD	−1SD	Normal

Psychiatric Diagnosis	ADHD-inattentive type	*Borderline* intelligence	Anxious disorder	*Borderline* intelligence	ASD (Asperger)	Anxiety disorder	Mood disorder
	Dyslexia	ADHD-impulsive type	Motor (graphic) delay	ADHD, ODD	Dysexecutive profile		Attachment disorder
	Pragmatic communication disorder	Dysexecutive profile		Visual-Spatial Dyspraxia	Dyspraxia		Written language difficulties
		At risk for dyslexia		Dysgraphia			NF1 related stress

Medication	Methylphenidate	Methylphenidate (not well tolerated)	Melatonin	Methylphenidate	Aripiprazole	None	Quetiapine
				Triptorelin Acetate			

Nonpharmacological treatment	Specific school adaptations	Specific school adaptations		Specific school adaptations	Specific school adaptations		
	Speech and reading therapy	Speech and reading therapy		Speech and reading therapy (past)	Language and motor remediation in the past		Speech and reading therapy
	Motor skills remediation	Motor skills remediation	Motor skills remediation	Motor skills remediation	Social strategies group		
			Psychotherapy	Psychodrama therapy		Psychotherapy	Psychiatric follow-up

Outcome	Improvement	Improvement	Stable	Improvement	Stable	Stable	Stable

ADHD, attention deficit hyperactivity disorder; ADI-R, autism diagnostic interview-Revised [15]; ADOS, autism diagnostic observation schedule [16]; ASD, autism spectrum disorder; BHK, concise evaluation scale for children handwriting [17]; EMOTION COMPREHENSION TEST, Test of emotion comprehension [27]; EXALANG, language evaluation for children 8–11 years [21]; NEPSY-II, Neuropsychological test for children [22]; NF1, Neurofibromatosis type 1; ODD, oppositional defiant disorder; TEA-Ch, test of everyday attention for children [24]; REY, Rey's complex figure test [25]; UBOs, unidentified bright objects]; WISC IV, the Wechsler intelligence scale for children [29].
